# Precise phase retrieval under harsh conditions by constructing new connected interferograms

**DOI:** 10.1038/srep24416

**Published:** 2016-04-14

**Authors:** Jian Deng, Dan Wu, Kai Wang, Javier Vargas

**Affiliations:** 1 Department of Electrical & Electronic Engineering, South University of Science and Technology of China, Shenzhen, 518055, China; 2 School of Electrical and Electronic Engineering, Nanyang Technological University, Singapore, 639798, Singapore; 3 Biocomputing Unit, Centro Nacional de Biotecnología-CSIC, C/Darwin 3, 28049, Cantoblanco (Madrid), Spain

## Abstract

To date, no phase-shifting method can accurately retrieve the phase map from a small set of noisy interferograms with low phase-shifts. In this Letter, we develop a novel approach to resolve this limitation under such harsh conditions. The proposed new method is based on constructing a set of connected interferograms by means of simple subtraction and addition operations, in which all the subset of interferograms have the same phase-shift interval of π/2. According to this characteristic, this set of connected interferograms can be processed with conventional phase retrieval methods as PCA or AIA obtaining accurate results. The reduction in the RMS errors after using our method reaches as high as 93.7% and 89.3% respectively comparing with conventional PCA and AIA methods under harsh conditions. Both simulation and experiment results demonstrate that the new proposed method provides an effective way, with high precision and robustness against noise, for phase retrieval.

Phase-shifting interferometry (PSI)^1^ is a high precision measuring technique of the optical phase, which has been widely used in various scientific fields as optical surface testing and deformation measurements^2^. Currently, several algorithms for phase and phase shift extraction from phase-shifted interferograms have been reported^3^,^4^,^5^,^6^,^7^,^8^,^9^,^10^,^11^,^12^,^13^,^14^,^15^,^16^,^17^,^18^,^19^,^20^,^21^,^22^ for various applications.

Advanced iterative algorithm (AIA)^3^, for example, is proposed to determine the phase shifts and the corresponding objective phase from a set of phase-shifted interferograms. Despite the highly accurate results provided by this approach, meanwhile it is highly time consuming due to iterative operation. An alternative method has been proposed by Hao *et al*.^4^ that retrieves the phase of each pixel by searching both the maximum and minimum intensity from a set of phase-shifted interferograms covering single phase-shifted period or more. However, this approach requires large quantity of interferograms to retrieve an accurate phase map. Other phase shift extraction methods proposed by Deng *et al*.^5^,^6^ are based on the Euclidean matrix norm and the 1-norm. Again, the same drawback comes up since the algorithms require a large number of interferograms, which should cover more than half of a phase shifted period.

In principle, the precision of phase retrieval algorithms is highly sensitive to the surrounding environment, in which undesired interference occurs constantly and in great extent. Moreover, the quality of the captured interferograms is highly sensitive to the performance of phase shifter, which means that in a practical PSI experiment, it is required to retrieve the phase from few phase-shifted interferograms with unknown phase shifts. Some approaches have been implemented to reduce the sensitivity to the environment. For instance, the “two-step” phase demodulation algorithms could be employed to reduce the influence of environmental factors (*e.g*. mechanical vibration and air turbulence). Other algorithms utilizing intensity difference of phase shifted interferograms^6^,^11^,^12^,^13^,^14^ could also retrieve the phase shift or the phase itself. Such algorithms can eliminate the background of the interferogram effectively hence retrieve phase with higher precision. In particular, Vargas *et al*.^15^,^16^,^17^,^18^,^19^ have proposed a new method based on principal component analysis (PCA) that is capable to extract the phase rapidly from series of randomly phase-shifted interferograms. Afterwards, Xu *et al*. has extended the PCA technique into multiple beam interferometry^20^ and then the method was improved by combining PCA and least squares approaches^21^ to retrieve phase with low residual phase error. Zhang *et al*.^22^ extended the PCA technique into simultaneous dual-wavelength phase-shifted interferometry. However, all of these presented PCA methods still require large number of phase shifted interferograms to maintain sufficient accuracy of phase retrieval.

All the above-mentioned phase demodulation algorithms would produce inaccurate results when using few phase-shifted interferograms with high noise level and small phase shifts. To solve these issues collectively, we propose a new method that would resolve these problems by constructing a new set of connected interferograms using simple subtraction and addition operations. The phase shifts of the new connected interferograms have special characteristics, which allow to retrieve the phase by conventional phase retrieval methods directly from these new connected interferograms.

## Methods

In PSI, the intensity distribution of one arbitrary pixel within the *n*th interferogram can be expressed as


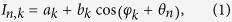


where *n* and *k* represent the sequence number of phase-shifted interferogram and the respective pixel position and *N* is the number of interferograms. Additionally, *a*_*k*_ and *b*_*k*_ represent the background and the modulation amplitude terms, and *φ*_*k*_ and *θ*_*n*_ are the objective phase and the phase shift of the *n*th interferogram. Finally, the total number of pixels composing an interferogram is *K*.

In our method, extra sets of interferograms could be constructed by subtraction and addition operations shown in Eqs. (2)–(4). A subtraction operation is first performed between interferograms to obtain intensity difference images by


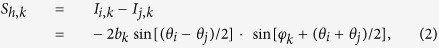


where *i*, *j* ∈ [1, *N*] with *i* < *j*. In Eq. (2), *h* is the sequence number of the intensity difference images, and *h* ∈ [1, *H*], with *H* the total number of the intensity difference images. It is easy to deduce that *H* = *N*(*N* − 1)/2. Then, an addition operation between interferograms is performed to obtain the intensity adding interferograms as


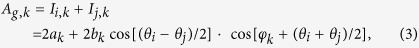


where *i*, *j* ∈ [1, *N*] with *i* < *j*, and *g* ∈ [1, *G*], the sequence number of the intensity adding images, where *G* is the total number of the intensity adding images. Again, it is easy to deduce that *G* = *N*(*N* − 1)/2. Note that *H* = *G*.

Combining the intensity difference with the intensity adding interferograms, we can construct a new connected interferograms set as


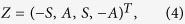


where *Z* is a matrix of size [2*N*(*N *− 1)] × *K*. These extra sets of connected interferograms could be post-processed to obtain the modulating phase, as demonstrated below.

Normalization is a key step of the proposed method. Moreover, in the process of normalization, Fourier transform method^23^ is one of essential and important steps. Normalization is performed from all the above connected interferograms. Methods of normalization have been reported in refs 24, 25, 26. The resultant normalized connected interferograms are denoted as


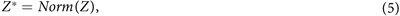


where *Norm*(·) represents the normalizing operator. For Eq. (1), the result of the normalized interferograms can be estimated as


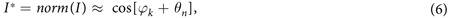


Similarly, *S*, −*S*, *A* and −*A* can be estimated as


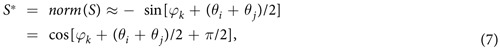



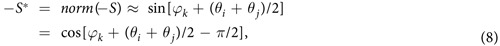



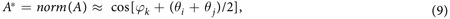



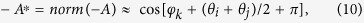


From Eqs. (7)–(10), it is easy to deduce that the normalized constructed images is also a set of phase shifted interferograms, with phase shifts of (*θ*_*i*_ + *θ*_*j*_)/2 − *π*/2, (*θ*_*i*_ + *θ*_*j*_)/2, (*θ*_*i*_ + *θ*_*j*_)/2 + *π*/2 and (*θ*_*i*_ + *θ*_*j*_)/2 + *π*. Given by the subtracting and adding operations between the interferograms, we have more interferograms with more different phase shifts. Using this characteristic, we can retrieve the objective phase easily and accurately from these new constructed images using phase retrieval methods, such as the PCA and AIA methods.

Our proposed method is based on three steps for phase retrieval. First of all, we use Eqs. (2)–(4), to construct the new connected interferograms set *Z*. Secondly, normalize these new connected interferograms to get the normalized interferograms Z*. Finally, by means of phase retrieval methods, such as PCA and AIA approaches, the phase map is obtained from these normalized interferograms.

## Results

**Figure 1:**
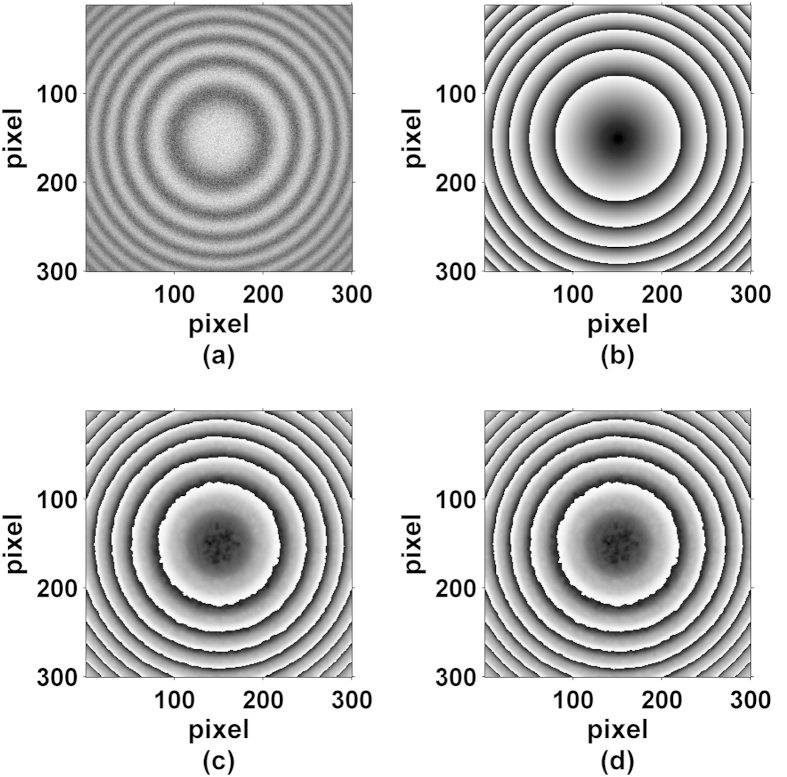
(**a**) One of simulated phase-shifted fringe patterns with strong noise (NSR: 30%), (**b**) the theoretical wrapped phase map, (**c,d**) are the wrapped phase map by PCA and AIA methods from the new constructed images.

To prove the effectiveness of the proposed method, we have tested it with simulated fringe patterns. In this simulation, only 8 interferograms are used to retrieve the phase. The phase shifts between fringe patterns are nonlinear and are respectively 0 rad, 0.10 rad, 0.17 rad, 0.20 rad, 0.24 rad, 0.32 rad, 0.35 rad and 0.4 rad. In addition, we have added some additive Gaussian noise to the interferograms with noise-to-signal ratio (NSR) of 30%. In Fig. 1(a), we show one of the simulated fringe patterns, in which the background and the modulation amplitude are respectively equal to 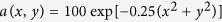 and 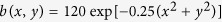. The size of the fringe pattern is of 300 × 300 pixels, and the phase corresponds to *φ*(*x*, *y*) = 4*π*[(*x*^2^ + *y*^2^)] − 〈4*π*[(*x*^2^ + *y*^2^)]〉, where <·> is the average operator, and −1.5 ≤ *x*, *y* ≤ 1.5. Figure 1(b) shows the theoretical wrapped phase map, and Fig. 1(c,d) present the obtained wrapped phase map by PCA and AIA methods from the new connected interferograms. Here, we term PCAN and AIAN to represent the new methods of phase demodulation by PCA and AIA methods from the new constructed connected interferograms respectively.

According to our proposed method outlined above, 112 new connected interferograms with different phase-shifts were constructed. To quantify the recovered phase error, we have computed the root mean square (RMS) error of the difference between the theoretical phase and the extracted one, which corresponds to 0.202 rad by PCAN method and 0.221 rad by AIAN method as shown in Table 1. The RMS errors by PCAN and AIAN methods are relatively close and small, indicating decent feasibility of our method in this difficult situation.

**Figure 2:**
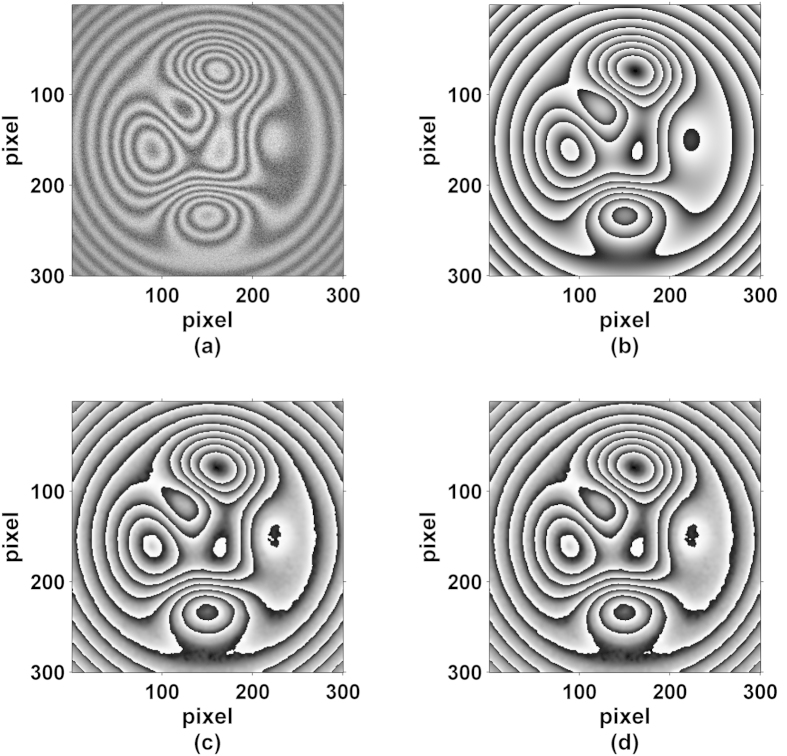
(**a**) One of simulated complicated fringe patterns, (**b**) the theoretical wrapped phase map, (**c,d**) are the wrapped phase map by PCA and AIA methods from the new constructed images.

**Figure 3:**
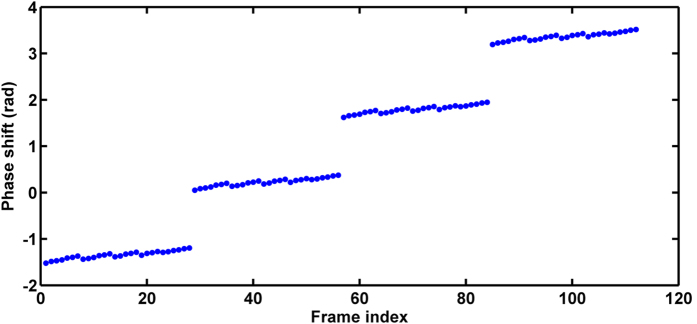
Phase shifts of the new constructed images.

Besides, we study the effect of complicated pattern on the measurement accuracy. As shown in Fig. 2(a), simulated fringe patterns with the size of 300 × 300 pixels are generated according to Eq. (1), in which the background and the modulation amplitude are the same as mentioned above. The measured phase distributions is set as the complex wavefront with 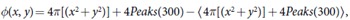 where “Peaks” is the peaks function in Matlab. The noise is also set as the additive Gaussian distribution with the NSR of 30%. Figure 2(b) shows the theoretical wrapped phase map, and Fig. 2(c,d) present the obtained wrapped phase map by PCAN and AIAN methods. The calculated results of RMS errors of the PCAN and AIAN methods are respectively 0.22 rad and 0.23 rad. The results indicate that the proposed method is suitable for complicated pattern. Besides, according to Eqs. (7)–(10), 8, 9, 10 and Fig. 3, the phase shifts of the new connected interferograms are distributed like a ladder and the phase shift interval between each subset image is π/2.

Moreover, another simulation was performed to study the effect of noise influence on the precision of retrieved phase. Table 1 provides the results that show the NSR of the interferograms ranging from 15% to 30%, with RMS errors from 0.155 rad to 0.202 rad by PCAN method, and from 0.160 rad to 0.221 rad by AIAN method. In Table 1, PCAN8 and AIAN8 represent phase retrieved by PCAN and AIAN methods from 8 original interferograms. It is therefore suggested that, by using PCAN and AIAN algorithms for different NSRs, these retrieved RMS errors would present very limited fluctuation indicating high robustness against noise.

From the results above, it is easy to indicate that our method present three advantages as can be used with: (1) small number of interferograms; (2) small phase shift interval between interferograms; (3) high level of noise. Since few original interferograms are used to construct extra sets of connected interferograms, our method generates more information for phase retrieval, hence presenting high robustness against noise. Besides, although the phase shifts of original interferogram is small, each subset images have the same small phase shift interval, and phase shift interval between each subset images is π/2, therefore, conventional phase retrieval methods can be used. Under these harsh conditions, especially in point (2) and (3), no other method can accurately retrieve the phase.

**Figure 4:**
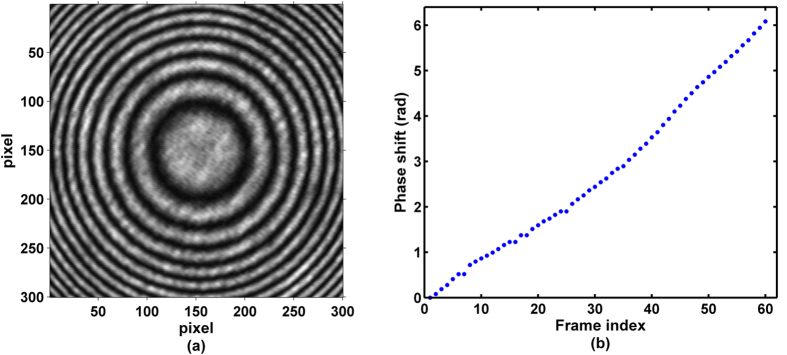
(**a**) One of experimental phase-shifted fringe patterns, (**b**) phase shifts of the captured interferograms.

To verify the above simulated results, the proposed method was also employed to perform phase extraction from experimental phase-shifted interferograms. In our experiment, 60 phase shifted interferograms were captured with size of 300 × 300 pixels. One example of the phase-shifted interferograms is provided in Fig. 4(a), and the phase shifts of the captured interferograms are shown in Fig. 4(b).

**Figure 5:**
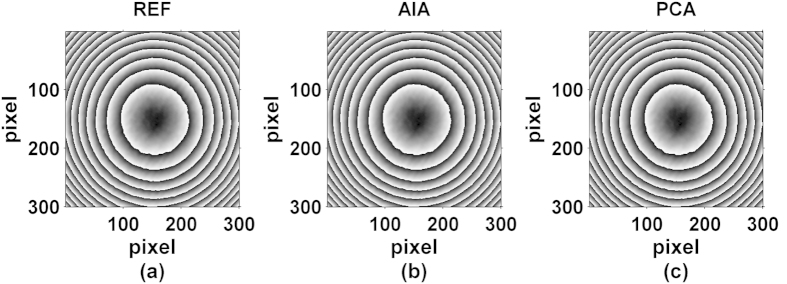
Reconstructed wrapped phase maps with different algorithms, (**a**) Four-step (REF), (**b**) AIA and (**c**) PCA.

We first obtained the wrapped phase map by the conventional four-step method using four phase shifted interferograms with respective phase shifts of approximately 0, π/2, π and 3π/2 rad, which was used as the reference phase, and this phase map is shown in Fig. 5(a). Later, alternative methods e.g. AIA and PCA were employed to obtain the same form of wrapped phase maps from all the 60 interferograms, as shown in Fig. 5(b,c).

**Figure 6:**
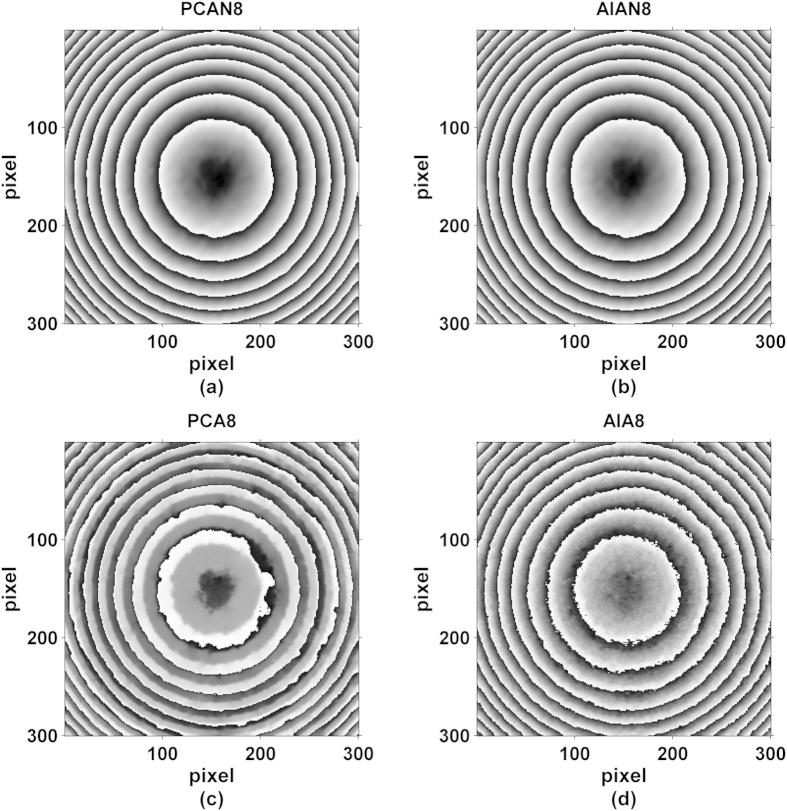
Reconstructed wrapped phase maps with different algorithms, (**a**) PCAN8, (**b**) AIAN8, (**c**) PCA8 and (**d**) AIA8.

**Figure 7:**
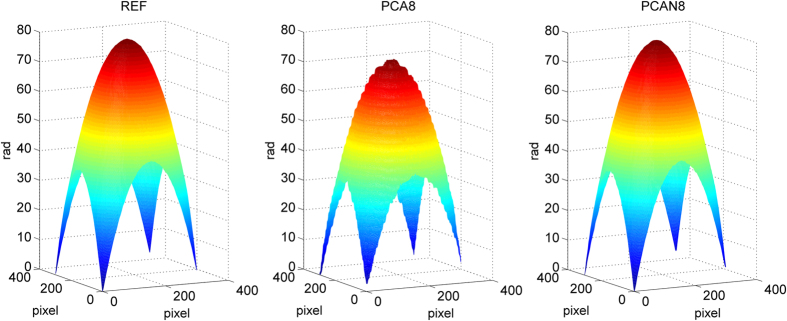
(**a**) Reference unwrapped phase map, and (**b,c**) are reconstructed unwrapped phase maps with PCA8 method and proposed PCAN8 method respectively.

**Figure 8:**
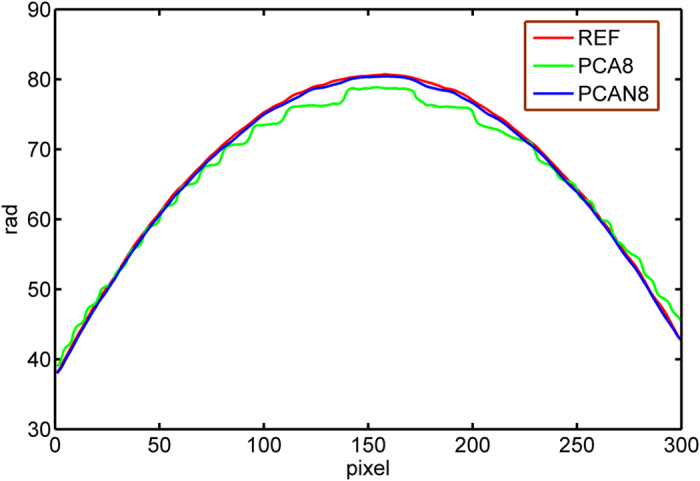
Phase distribution of the interferogram cross-section, where red line is the reference value, green line is obtained by PCA8 method and blue line is obtained by PCAN8 method.

The performance of our proposed method is also shown superior, especially for the case of using few interferograms with small phase-shifts. For comparison, we selected only 8 interferograms from the first of 60, and successfully obtained a new set of phase maps using the PCAN and AIAN methods versus PCA and AIA methods, as provided in Fig. 6(a–d) and written as PCAN8, AIAN8, PCA8 and AIA8. Here, PCAN8 and AIAN8 represent the phase retrieved by PCAN and AIAN methods from 8 original interferograms, and PCA8 and AIA8 represent the phase retrieved by PCA and AIA methods from the 8 interferograms from the first of 60. Unwrapped phase maps using four-step method (as reference), PCA8 method and PCAN8 method are shown in Fig. 7. Comparing to the reference phase map, it is clearly shown that the phase obtained by PCA8 method appears serious distortion, while the phase obtained by PCAN8 method is nearly the same as the reference. Figure 8 shows the phase distribution of the interferogram cross-section, where red line is the reference value, green line and blue line are the phase of cross section by PCA8 method and PCAN8 method respectively. It is confirmed that the phase of cross section obtained by PCA8 method suffers obvious deviation, while that by PCAN8 method is agreed well with the reference, which indicates that precision of PCAN8 method is much higher than that of by PCA8 method under this harsh condition.

In addition, the RMS errors of all the methods with respect to reference are provided in Table 2. Clearly, the AIA and PCA methods using 60 (all) interferograms shows very small RMS errors, indicating great precision of their calculated results that could be ultimately achieved by excessive number of interferograms.

However, the precision of phase extraction using the same methods but with only 8 interferograms is rather low, giving RMS errors 1.162 rad and 1.887 rad respectively for AIA8 and PCA8. In contrast, the proposed methods of PCAN and AIAN have demonstrated relatively small magnitude of RMS error as 0.118 rad and 0.124 rad respectively, which is far below than that of AIA8 and PCA8 under the same condition with reduction of 93.7% and 89.3%, hence suggesting high robustness of the proposed method precision against limited number of interferograms with small phase shifts. The main reason for such advantage is that the phase shifts between interferograms are small. Therefore, the proposed method could be more preferable over AIA and PCA methods in case of using few interferograms with small phase shifts. Another advantage of our method is the low sensitivity against noise, since it could also give decent performance from noisy interferograms with the uncertain phase shifts as illustrated in Fig. 3. It is therefore suggested that our method could be also applied under noisy condition.

## Discussion

**Figure 9:**
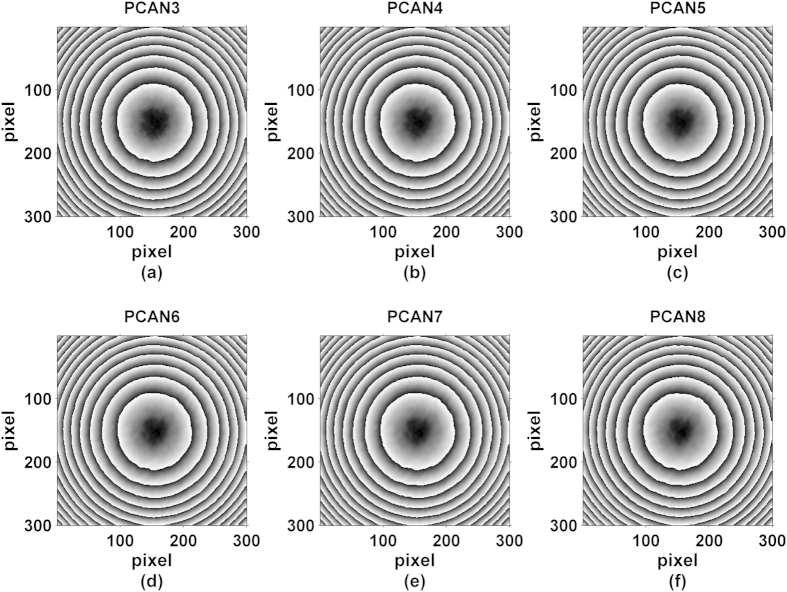
Reconstructed wrapped phase maps with PCAN algorithm using different number of phase shifted interferogram.

To analyze the influence of using different numbers of interferograms with our proposed method, we have separately performed the phase retrieval with the first 3, 4, 5, 6, 7 and 8 interferograms. In Fig. 9, the obtained wrapped phases map e.g. PCAN3 refers to phase map obtained from 3 phase-shifted interferograms, and so forth. The RMS errors of PCAN3 to PCAN8 are shown in Table 3, in which all of them are close to 0.12 rad that suggests almost the same precision regardless of the number of interferograms used.

In practical applications, our method would be preferable since fewer number of phase shifted interferograms (typically around 3~5 frames) could minimize the effects of vibrations, as well as saving the processing time. However, if the interferograms are found noisy in great extent, then using more phase shifted interferograms to retrieve phase would help improve the precision. Obviously, in this case, it is better to use more frames than 5.

Besides, we have tested other linear combinations to construct a new connected interferograms set to retrieve phase. Assume that *Z*_1_ = (−*S*, *A*, *S*, −*A*, *I*, −*I*)^*T*^and*Z*_2_ = (−*S*, *A*, *S*, −*A*)^*T*^, here *Z*_2_ is the same as Z mentioned above, the simulation and experiment calculated data is the same as the calculated data of Figs 1 and 4. The calculated results are obtained by PCAN method. For simulation case, using Z_1_ to retrieve phase, the RMS error is 0.219 rad, while using Z_2_ to retrieve phase, the RMS error is 0.202 rad. For experiment case, using Z_1_ to retrieve phase, the RMS is 0.122 rad, while using Z_2_ to retrieve phase, the RMS is 0.118 rad. From both the simulation and experimental results, it is clearly to indicate that using Z_2_ is slightly better than Z_1_. The main reason is that, for Z_2_, the phase shifts distribution are better than Z_1_ in the range of [0, 2π]. In other words, using Z_2_ to retrieve phase, the data is more satisfied the condition of using PCA method. Actually speaking, from the view of input information, Z_1_ is more than Z_2_, but phase shift of A is *θ*_*i*_ and −*A* is *θ*_*i*_ + *π*, if we added another set of interferograms B and phase shifts of B is *θ*_*i*_ + *π*/2 as well as −*B* is *θ*_*i*_ + 3*π*/2, and construct a new set *Z*_3_ = (−*S*, *A*, *S*, −*A*, *I*, −*I*, *B*, −*B*)^*T*^, in this case, the obtained result is better than Z_2_.

**Figure 10:**
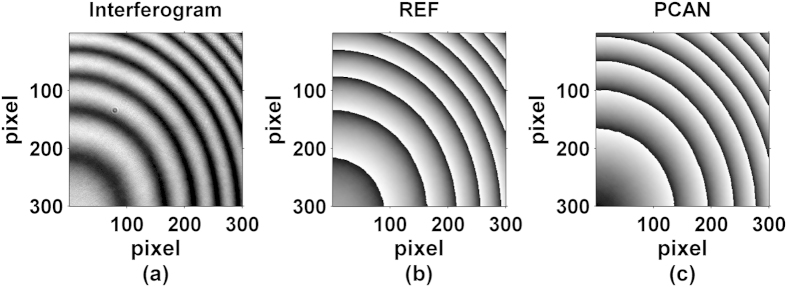
(**a**) One of experimental complicated fringe patterns, (**b**) the reference wrapped phase map, (**c**) the wrapped phase map by PCA methods from the new constructed images.

Finally, we have tested another interferogram with irregular fringe pattern. As shown in Fig. 10, Fig. 10(a) shows the interferogram with irregular fringe pattern, Fig. 10(b) is the reference wrapped phase, and Fig. 10(c) is the obtained wrapped phase map by PCAN method. The RMS error of the PCAN method is 0.13 rad. The result indicates that the RMS error is small and the proposed method is also suitable for complicated pattern.

## Conclusion

In summary, to date, no other method can retrieve phase accurately under these harsh conditions, such as: (1) a small number of phase-shifted interferograms; (2) the interferogram with strong noise; (3) a very small phase shift interval between interferograms. In this paper, we propose a new method that would resolve these difficulties. The proposed method skillfully constructs a set of new connected interferograms by means of simple subtraction and addition operations, in which each subset images has the same small phase shift interval, but phase shift interval between each subset images is π/2. Using this characteristic and combining with some conventional phase retrieval methods, such as PCA and AIA methods, phase can be retrieved easily from these new constructed images. According to the simulated and experimental results, it is proved that the phase can still be obtained with high precision under these harsh conditions.

## Additional Information

**How to cite this article**: Deng, J. *et al*. Precise phase retrieval under harsh conditions by constructing new connected interferograms. *Sci. Rep*. **6**, 24416; doi: 10.1038/srep24416 (2016).

## Figures and Tables

**Table 1:** RMS Errors Of Phase Extraction With PCAN and AIAN Algorithms By Interferograms With Different Noise.

NSR	15%	20%	25%	30%
RMS_PCAN8 (rad)	0.155	0.169	0.185	0.202
RMS_AIAN8 (rad)	0.160	0.177	0.197	0.221

**Table 2:** RMS Errors of Phase Extraction With Different Algorithms.

	PCAN8	PCA60	PCA8	AIAN8	AIA60	AIA8
RMS (rad)	0.118	0.020	1.887	0.124	0.015	1.162

**Table 3:** RMS Errors Of Phase Extraction With PCAN and AIAN Algorithms By Different Number Of Interferogram.

Number	3	4	5	6	7	8
RMS_PCAN (rad)	0.126	0.120	0.118	0.117	0.119	0.118
RMS_AIAN (rad)	0.126	0.120	0.118	0.117	0.128	0.124
